# EXPERIENCES OF CHAIN OF CARE AND REHABILITATION AFTER STROKE: A QUALITATIVE STUDY OF PERSONS DISCHARGED TO SKILLED NURSING FACILITIES BEFORE RETURNING HOME

**DOI:** 10.2340/jrm.v56.35240

**Published:** 2024-06-20

**Authors:** Sofie FORS, Anna BRÅNDAL, Hélène PESSAH-RASMUSSEN, Ingrid LINDGREN

**Affiliations:** 1 Department of Neurology, Rehabilitation Medicine, Memory Disorders and Geriatrics, Skåne University Hospital, Sweden; 2 Department of Community Medicine and Rehabilitation, Physiotherapy, Umeå University, Sweden; 3 Department of Clinical Sciences, Lund, Lund University, Sweden; 4 Department of Health Sciences, Lund University, Sweden

**Keywords:** stroke, rehabilitation, skilled nursing facility, independent living, interviews

## Abstract

**Objective:**

To explore how people with stroke, discharged to skilled nursing facilities before returning home, experience the chain of care and rehabilitation.

**Design:**

Qualitative, semi-structured interview design.

**Methods:**

Thirteen stroke survivors discharged from a stroke unit to a skilled nursing facility before returning to independent living participated. Semi-structured telephone interviews were conducted 2–5 months after stroke and analysed with content analysis.

**Results:**

The analysis resulted in three categories, *Organizational processes, critical and complex, Rehabilitation, the right support at the right time* and *Adaptation to the changed situation*, with a total of 9 subcategories. The informants perceived low participation in planning and goalsetting and limited information. Support from the healthcare services was important to proceed with improvements although the amount of supported training varied. Factors hindering and facilitating managing everyday life were described, as well as lingering uncertainty of what the future would be like.

**Conclusion:**

Support and rehabilitation as well as individuals’ needs varied, throughout the chain of care. To enable participation in the rehabilitation, assistance in setting goals and repeated information is warranted. Tailored care and rehabilitation throughout the chain of care should be provided, followed up at home, and coordinated for smooth transitions between organizations.

LAY ABSTRACT

A stroke can affect various aspects of daily living. After acute care, many people need rehabilitation in different settings to become as well as possible. This study sought to explore how people with stroke, discharged to skilled nursing facilities before returning home, experience the chain of care and rehabilitation. Their experiences could help improvement in rehabilitation services. The results showed that those with stroke did not feel involved in the planning, and information got lost. The amount of rehabilitation and support with training varied. Uncertainty regarding the future was expressed. To make the stroke survivor more involved and secure, routines for continuity in rehabilitation throughout the chain of care need improving and relevant information should be given. Individuals handle the changed situation differently. Support should be offered in adapting and managing everyday life as long as needed.

A stroke can affect various aspects of daily living. For many people, rehabilitation to improve functioning, participation, and well-being might be needed (1). According to the WHO (World Health Organization), the definition of rehabilitation is “a set of interventions designed to optimize functioning and reduce disability in individuals with health conditions in interaction with their environment” (2). Rehabilitation efforts include a combination of *training*, *elimination* and *compensation*. *Training* is carried out with the aim of improving an activity or a function. *Elimination* of symptoms can be medical treatment and *compensation* is an adaptation to a disability, for example with aids (3). Regarding training, there is a time-limited window of neuroplasticity during which it has the greatest function-enhancing effect (4).

In Sweden, several different care providers are involved in care and rehabilitation after stroke (5). Receiving organized care in a stroke unit is highly valuable (6). Rehabilitation often starts at the stroke unit. However, during the last decade, hospital stay at the stroke unit has decreased in Sweden, and is now on average 7 days (7). Related to the individual’s function and level of dependence, the rehabilitation can proceed in different organizations after the stroke unit care. The skilled nursing facility (SNF) is an important part of the chain of care for people with more extensive care and rehabilitation needs who are not able to be discharged home directly. SNF is a time-limited, care-need assessed, nursing and inpatient rehabilitation centre. It is usually staffed with assistant and registered nurses as well as physio- and occupational therapists, trained to help people with various diagnoses and needs (8). When support from the municipality is needed after discharge from hospital, the municipality independently decides how the rehabilitation and care is organized. Duration, frequency, and choice of training activities is not predefined, but usually based on the person’s individual needs and goals. The rehabilitation consists of improving functions or finding aids for managing activities of daily life (9). The period at SNF is often determined for about 2 weeks with a possibility to extend, based on the need for care. The decision on extension is made by an official (9).

Experiences from people with stroke, discharged home directly after stroke unit care, have been well explored (10–17). However, experiences from those discharged to an SNF before returning home have to our knowledge not been studied. As this is a well-established care pathway in the Swedish context, and similar pathways may exist in other countries, it is important to gain insight into how these people experience their care and rehabilitation. This study therefore aimed to explore how people with stroke, discharged to skilled nursing facilities before returning home, experience the chain of care and rehabilitation.

## METHODS AND MATERIALS

### Design

A qualitative semi-structured interview design was used (18). Content analysis (19) of telephone interviews was conducted. The Consolidated Criteria for Reporting Qualitative Studies (COREQ) guide was followed (20).

### Study context

**Fig. 1:**
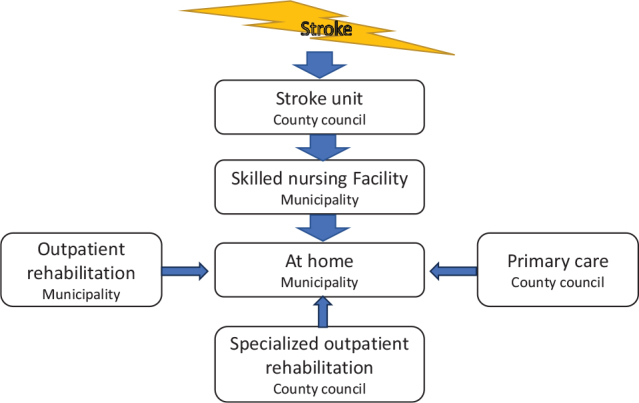
Description of responsible organization as well as the chain of care and rehabilitation for the study.

In the area where the study was conducted (southern Sweden), the county council is responsible for specialized in- and outpatient rehabilitation in hospital, and for primary care (5) while the municipalities provide care and rehabilitation at home and at nursing facilities (SNF) (5). The chain of care for this study is pictured in Fig. 1.

### Participants and sampling

Inclusion criteria were: 2 to 5 months since stroke onset, discharged to an SNF before returning home, and verbally proficient in Swedish. Exclusion criteria were: aphasia, cognitive impairment, or comorbidity that would prevent participation in interviews.

Possible participants were identified by 2 of the authors (SF and HPR) through contacts at the stroke units at Skåne university hospital who continuously registered the patients with stroke who were discharged to an SNF. The local register of the Swedish Stroke Register, Riksstroke (21), was also used for retrospective identification. Purposive sampling was used (18). Individuals who met the criteria were contacted by telephone and informed about the study. Written information and a consent form were sent to those who were interested. A week later, they were contacted again regarding interest in participation. Participant demographics were obtained from medical records and the interviews.

### Materials

All authors were involved in developing the interview guide (see Appendix S1). Key areas were investigated through a literature search of rehabilitation after stroke. Topics such as transitions between care units, rehabilitation in different settings, participation in setting goals, and homecoming were identified. To collect background information on the informants, the interview guide also included questions from the Post Stroke Checklist – 14 items (22). The guide was pretested on 2 people by the first author, thereafter discussed with the last author, and revised.

All authors have long (more than 10 years) experience of rehabilitation after stroke. Three have long experience in research (AB, HPR, IL), 2 of them in qualitative research (AB, IL).

### Assessment instrument for background data

Based on the interviews, the Modified Rankin Scale (mRS) was used to grade the participants’ functional level of independence. It is a single-item, global outcomes rating scale for patients post-stroke with reference to pre-stroke activities. The scale ranges from 0 (no symptoms) to 6 (dead) (23).

### Data collection

Semi-structured interviews were conducted by telephone, by the first author (SF), who had no previous connection to the study participants. Probing questions were used to deepen interesting topics. The interviews were recorded as audio files and transcribed verbatim by the first author. Their length varied between 30 and 71 min (Mean [SD] 46 [14] min).

### Data analysis

Latent content analysis according to Graneheim and Lundman was used (19). After transcription, the interviews were read thoroughly by the first author to gain an impression of the content in its entirety. Meaning units, segments that conveyed interesting information in relation to the aim of the study, were derived from the transcript, condensed, and given codes capturing the key concept of the text. The codes were abstracted and sorted into subcategories and main categories based on how they related, by 3 of the authors (SF, AB, IL) (see Table I for an example). Citations for each subcategory were derived from the transcripts.

**Table I:** Example of the organizing phase with open coding, abstracting and creating categories

Codes	Subcategory	Main category
Hospital – short stay # 10 Passive – do as they say # 5 SNF – no plan before discharge # 6 Hospital – no conversation regarding home planning # 4	Inadequate routines and low participation in rehabilitation planning	Organizational processes, critical and complex
Disappointed, not knowing time limit on home rehabilitation # 1 Do not know how to train #4 No information about stroke preventive lifestyle changes # 1–6, 8, 9	Lacking information creates uncertainty	
Weird feeling returning home # 1 Worried about managing at home # 9 Good to be home # 11	Homecoming, desired but sometimes daunting	

SNF: Skilled nursing facility.

### Ethical considerations

The Swedish Ethical Review Authority approved the research (Dno: 2021-02425 and DNR 2019-01044). The study followed the ethical requirements of the Declaration of Helsinki (24). Informed written consent was obtained. All participants were informed that their participation was confidential, voluntary, and could at any time be withdrawn. If further rehabilitation was requested, the participant was offered support in getting help. Interviews were coded to prevent individuals from being identified. The data are stored inaccessibly to unauthorized personnel.

## RESULTS

Thirteen participants, 6 of them men, were interviewed from October 2021 to August 2022, 2–5 months after stroke. Their ages varied between 59 and 92 years (Mean [SD] 77 [6] years). Nine out of 13 were dependent in daily activities and used walking aids. Informant characteristics are indicated in Table II.

**Table II:** Characteristics of informants at the time of the interviews

Informant	Sex	Age	Social status	mRS
1	Female	75	Living alone	3
2	Female	86	Living alone	3
3	Male	74	Living with spouse	3
4	Male	72	Living with spouse	2
5	Female	87	Living alone	3
6	Male	78	Living with spouse	3
7	Female	68	Living alone	1
8	Male	80	Living alone	3
9	Female	92	Living alone	3
10	Female	80	Living alone	3
11	Male	74	Living alone	3
12	Female	75	Living alone	3
13	Male	59	Living alone	3

mRS: Modified Rankin scale.

**Fig. 2:**
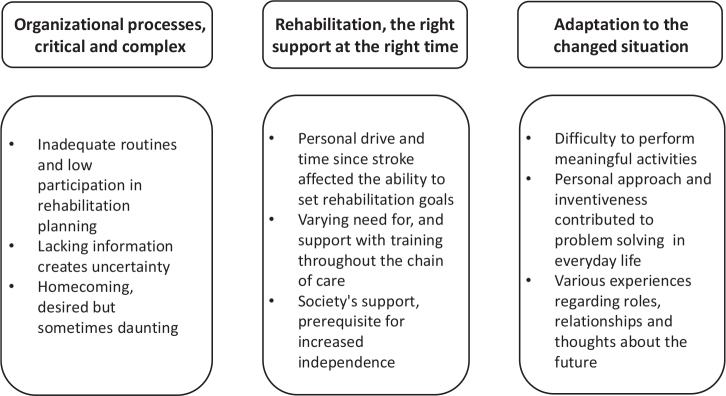
Results with categories and subcategories.

The analysis resulted in 3 categories, *Organizational processes, critical and complex, Rehabilitation, the right support at the right time*, and *Adaptation to the changed situation*, with a total of 9 subcategories, which describe different aspects important for improvement. The results are presented in Fig. 2 and further described under separate headings below.

### Organizational processes, critical and complex

This category comprised descriptions of sometimes inadequate routines for rehabilitation planning with experiences of low participation, and a lack of both medical and practical information. Also described were mixed feelings about coming home, longing for the familiar but also uncertainty as how to to manage.

### Inadequate routines and low participation in rehabilitation planning

The time in hospital was short and blurry and many described adopting a passive role while subject to the examinations they were told to undergo. Few informants experienced taking part in their rehabilitation planning either before or at the SNF. The routines regarding referrals to mobility services or further outpatient rehabilitation after SNF were described by some informants as deficient, resulting in delayed training.

But it [the referral to the municipal outpatient rehab centre] was not done, it had not been sent. So now it was delayed a couple of months because of that, and the same with that mobility service … they forgot to give that form when I got there [to the SNF]. And you should get it as soon as you get there so you can sign if you need to, because it’s such a long wait. (#1)

### Lacking information creates uncertainty

Most of the informants described lacking information in multiple areas, both medical and practical. Regarding stroke consequences, some lacked information concerning fatigue. Moreover, information on lifestyle factors for stroke prevention was rarely received. Uncertainty regarding practical information described by the informants related, for example, to costs and duration of interventions and waiting time for rehabilitation.

Because you get 2, from the municipality, 2 weeks I think … that I got stair climbing exercise. I didn’t know that it was only 2 weeks so it was like a long … I got…. I felt really disappointed because it’s better to know than him saying “now it was the last time”. And I had so looked forward to the next time. (#1)

### Homecoming, desired but sometimes daunting

Most agreed that coming home was comforting. Many longed to see their pet or partner again, but some also experienced anxiousness. They described concerns regarding leaving the SNF where they were monitored, and uncertain whether they would be safe at home. But a majority described how they received aids and other adaptations immediately after leaving the SNF, which facilitated their homecoming. One informant said that when she got home, she felt safer than expected thanks to the many visits from the municipality home rehabilitation team.

I actually thought I would be very confused [coming home] but I wasn’t at all. Because there were so many people who met up with me. And I wasn’t particularly homesick because I was mostly afraid of how I would cope. But it has gone great. (#9)

### Rehabilitation, the right support at the right time

The need for support with setting rehabilitation goals and training to reach them differed. Depending on the municipality in which the informant lived, so did the possibility for receiving supported training in SNF and afterwards at home. Home care support and accessibility to aids were described as vital to progress. The care was also affected by informants’ experience of how they were personally treated by the personnel.

### Personal drive and time since stroke affected the ability to set rehabilitation goals

The ability to set rehabilitation goals varied among the informants. Many expressed themselves vaguely regarding their goals, while others were more specific, for example to be able to use the toilet on their own or to be self-reliant at home. One informant said that she immediately had her goal clear, returning home! Some informants described difficulties setting goals early on when the prognosis was uncertain. What could be realistic goals appeared clearer to them as recovery proceeded. Only one informant described getting help in setting goals.

When they asked me about it [goals], very early on, then I thought it was more difficult … but if you ask me today there are no doubts about what my goals are…. Goals, it’s not that hard to set goals, really, but the question is if you can fulfil them. (#4)

### Varying need for and support with training through-out the chain of care

All informants spoke of their progress but the experience varied as to where, in the chain of care, the improvements began or culminated. Some described remission of symptoms at the hospital, some “revelational improvements” at the SNF, while many said that things had developed after coming home. Also, the support with training differed. Few recollected any training at the hospital; most said that the training started at the SNF. The time spent at SNF was mainly described as valuable, although some experienced it as inadequate with very little training or not adjusted to the challenges in everyday life. At home, the time with supported training varied from twice a day to once a month. Sometimes supported training was less than promised due to lack of time from the personnel.

I basically have nothing [help with training at home], but it is as X [caring personnel] says, she is doing the best she can. But I have to say that they are really overwhelmed with work, these poor personnel. They are so talented. She’s trying to fit me in, will try today. She has 3 times [a week] taken me for a walk, however she’ll manage that…. I think it’s 3 times I have [been granted], but it doesn’t turn out so that often. (#1)

### Societal support, a prerequisite for increased independence

Support from society such as home care, aids, mobility services, and healthcare transport was described as a prerequisite for independent living and proceeding with improvements after SNF. Most said that the aids and housing adaptations were sufficient, although some were still waiting for the adaptations to be made. To reclaim activities and get to the municipal outpatient rehab centre, the mobility service was valuable. However, processing time was long, and the mobility service was sometimes unreliable and seldom as efficient as the informants wanted. Long travel time and multiple stops were common. Also, one woman was left outside her building not able to get inside on her own.

… and then I take a mobility service when I’m going over to someone. It works reasonably well. Some drivers have no empathy, on 2 occasions they have left me at the pavement and said, this is fine, and then they drove away. I have 5 steps to climb and a gate to open. And the fourth floor, with an elevator though. But we complained about that. I sat on the street until someone came that I could ask for help. (#2)

The informants stated that not only practical support, but also mental support was needed. The care providers at the hospital were described as supportive, mitigating homesickness, and homecare personnel as caring and a “psychological support”. However, some informants experienced less empathetic personnel which affected both their motivation and further care.

When I was at X [the SNF], I had no problems with bladder control … at X [the SNF] there was one night that I called [for the personnel]…. I needed to go to the toilet…. It took more than half an hour [before someone came]. I was in so much pain that my entire lower abdomen burned, and I kicked the bed because of it. I needed to pee so much. And then they came, the girls. And one was so annoyed with me, the other she said nothing. And I could hardly pee. And then in the afternoon they had to give me a catheter … one of them, she was so annoyed, blamed it on that they had so much to do. Then I had to have a catheter for 14 days–3 weeks. (# 9)

### Adaptation to the changed situation

Even if several months had passed since the stroke, and the informants had returned home, all spoke about difficulties in everyday life as a result of their stroke. Personal drive and inventiveness affected the ability to handle these difficulties. For many, relationships and roles had changed, and the future felt unclear.

### Difficulty in performing meaningful activities

The majority reported difficulty participating in activities that were meaningful for them before the stroke. Bridge was played in a cellar with steep stairs, an outdoorsy person had trouble leaving home, and many felt isolated not being able to drive. Having friends and relatives close by was described as positive. Not being able to participate in activities was perceived to have consequences for social life.

It will be an issue if I won’t be able to do it [play golf] again because it will change my life quite a lot. Everyone I hang out with, everyone we hang out with, plays golf. *laughs* It’s that we play a lot of pairs golf. My wife plays, she plays with other couples and such. And if I will not be able to play anymore, there would be quite a big change in our social life. (#4)

### Personal approach and inventiveness contributed to problem-solving in everyday life

The informants experienced physical symptoms like lack of motor control, spasticity, and postural instability as well as mental and cognitive difficulties such as problems with memory, attention, and fatigue. Also described were worrying thoughts and uncertainty regarding the future. A positive attitude, industriousness, tenacity, and inventiveness were described as facilitating factors for handling the everyday challenges.

It’s tough to only have one working hand. Then you have to be an inventor. But it’s not a problem, it is, so to speak, it was a challenge just to put toothpaste on the toothbrush. How to do things if you only have one working hand. But *laughs* you just have to keep on working. (#8)

### Various experiences regarding roles, relationships, and thoughts about the future

Many described great support from relatives and friends to adapt, practical help with both chores and training, and emotionally dealing with worries concerning the future. However, some made clear their relatives’ help was not to be expected. Relationships sometimes changed and became more complicated as the informants became more dependent on their next of kin for help. Despite support and improvements, the informants still pondered about how far recovery would take them and what their role would be in the future.

It’s like this: to get well or not to get well? And how long will it take? It’s actually quite enervating. You think about that a lot, right. (#6)

## DISCUSSION

The aim of this study was to explore how people with stroke, discharged to skilled nursing facilities before returning home, experienced the chain of care and rehabilitation. Three main categories emerged in the analysis, *Organizational processes, critical and complex*, *Rehabilitation, the right support at the right time*, and *Adaptation to the changed situation*. The informants perceived low participation in planning and goal-setting and limited information. Support from the healthcare services was important to proceed with improvements although the amount of supported training varied. Factors hindering and facilitating managing everyday life were described, as well as lingering uncertainty as to what the future would be like.

In transitions to and from the SNF, our informants describe low participation in care and rehabilitation planning. Low participation has been reported before (10) but also contradicted (11). A study from 2004 described how care and rehabilitation planning took place at the hospital before discharge and that relatives also could participate (11). Since then, hospital stay has shortened and care planning at hospitals in Sweden has been replaced by digital contact between care providers (7, 25, 26). A consequence of shorter length of stay at the stroke unit might be that information is merely forwarded to the receiving care provider, without sharing with the individual. When implementing a new system for planning between different care providers, it is important to ensure patient participation. Moreover, our informants described obstacles to continuity in the rehabilitation process between the transitions, with waiting periods and missing referrals. Deficiencies in patient support during transitions between care units have previously been reported (27) and indicate that the work with seamless transition processes is not sufficient. Also described by our informants was a lack of information, which can create stress when returning home (14). To reduce the risk of information falling through the cracks during transitions (28), we see a need for a more transparent means of communication and coordination between care providers. One approach to make the patient more involved in the planning and process, and to ensure smooth transitions, can be a written document describing goals and agreements between the person and the care providers. This should also include a named professional who can be contacted with questions (29).

Returning home can be a big step in the rehabilitation process but may also create insecurity (11, 12, 14). However, our informants did not mention a feeling of abandonment (27) and it is possible that discharge to an SNF enables preparation time before returning home. But, in accordance with a previous study, our informants expressed how professionals’ visits after discharge enhanced the sense of security (12). This highlights the importance of follow-up and support after discharge, even after passing through the SNF.

Time to adapt was perceived as important for the ability to set goals. The difficulty of setting goals early on could probably relate to the perceived passivity in the hospital and lack of awareness of prognosis (30, 31). The time at the SNF could help the person become more active in the planning and take back control of their situation, but support from professionals might be needed to set realistic goals (30).

The amount of rehabilitation received varied among our informants, both at the SNF and after returning home. This is in accordance with an earlier report (13) and stroke survivors often experience a greater need for rehabilitation than they are offered (32, 33). Also, when the person is moved from a unit with extensive stroke knowledge to the municipal organization where professionals usually have more general knowledge, the focus might change. Both a rehabilitation approach and specific stroke knowledge are needed to ensure the right kind, amount, and duration of training to optimize individual function. If rehabilitation consists of, mainly, early compensation strategies at the price of less training, there is a risk of people being more dependent than necessary, leading to a larger cost for society (34). Moreover, the healthcare planning is not always in line with the recovery process of the individual. Individuals might be waiting for adequate interventions when function-enhancing training would be most effective (4). Although societal support was often enough, some had to wait for the right adaptations or assistance with mobility service. Waiting periods delay returning to everyday life, meaningful activities, and participation in society, which are the main aspects for rehabilitation.

Regarding the category *Adaptation to the changed situation*, environmental factors such as closeness to friends and family were described as beneficial for facilitating participation in society (35). A limiting environmental factor described by our informants was the driving ban (36). Early access to mobility services can facilitate access to rehabilitation, friends, and meaningful activities. Leisure and social activities are important parts of recovery (36), but were described by our informants as difficult to perform. It is possible that our informants were still at an early stage in the recovery process and maybe more focused on intense training to regain function (15, 16). The early stage could also explain that the future was not yet clear and certain. Also attitude and inventiveness affected how to manage everyday life. It seems that people have different prerequisites to retake control. This indicates that support in rehabilitation and follow-up needs to be offered as long as there is a requirement after the stroke unit care.

The informants spoke of the significance of relatives in managing everyday life, but also that their help could not automatically be expected. Most of the informants in this study lived alone, which in Sweden is common among older people (37), and may increase the likelihood of discharge to an SNF after stroke unit care (38). An important consideration when planning for rehabilitation should be to support the person in being able to manage rehabilitation, even without the support of relatives.

### Strengths and limitations

A strength of our study is that participants were included from hospitals in 2 different cities, from multiple SNFs, were of both sexes, with different social status and with varied disabilities. Participants with various experiences increase the possibility of gaining wider knowledge regarding the research question, improving credibility (19). Interviews were held over the telephone because of restrictions due to COVID-19. The use of telephone calls in interview situations has been utilized with good results (39), but might impede communication and obstruct detection of fatigue.

Regarding dependability, the interview guide was created according to areas described as important in previous studies. It was pretested, discussed within the authors, and adjusted before use. The interview guide is included as Appendix S1. In the analytic process, the subcategories were discussed repeatedly among the authors and quotations were inserted in the results. A limitation might be that the interview guide consisted of many questions, which may have resulted in brief answers. Moreover, the participants have not taken part in the content of the transcribed material, which prevents clarification of statements. Prior understanding regarding stroke rehabilitation can be both a strength and a weakness. All researchers had many years of experience from working with rehabilitation after stroke, which can affect the interpretation of the results. The interviewer had limited experience in interview techniques and greater experience may have led to other follow-up questions and more detailed answers.

The results share experiences of stroke care in the area where the study was conducted. Due to the design of the study, individuals with aphasia or severe cognitive impairments, who were unable to participate in an interview, were excluded. We consider the results to be transferable to people in similar conditions and contexts, but not to be generalized to all stroke survivors.

### Implications for practice

This study contributes to a deeper understanding of experiences of rehabilitation throughout the chain of care including transitions to and from an SNF, as well as how remaining symptoms affect and are managed by the person at home. This knowledge can help care providers to streamline individualized rehabilitation. From the results of this study, we see the importance of providing rehabilitation and information throughout the chain of care with communication between the county council and municipality, to avoid gaps in the rehabilitation after stroke. Participation in the rehabilitation planning is important and should be ensured, preferably with decisions compiled in a written document to reinforce recollection. Support and follow-up should be offered until the individual is back in control of the situation.

Wider research advocates the need for a closer look at organizational processes that hinder or facilitate smooth transitions. Focus groups with care providers from various links within the chain of care can discuss what is required for better collaboration and smoother care transitions.

In conclusion, support and rehabilitation as well as individuals’ needs varied throughout the chain of care for stroke survivors discharged to skilled nursing facilities before returning home. To enable participation in their rehabilitation, assistance in setting goals and repeated information is warranted. Tailored care and rehabilitation throughout the chain of care should be provided, followed up at home and coordinated for smooth transitions between organizations.

## Supplementary Material

EXPERIENCES OF CHAIN OF CARE AND REHABILITATION AFTER STROKE: A QUALITATIVE STUDY OF PERSONS DISCHARGED TO SKILLED NURSING FACILITIES BEFORE RETURNING HOME
