# Supplementary material for: EXPERIENCES OF CHAIN OF CARE AND REHABILITATION AFTER STROKE: A QUALITATIVE STUDY OF PERSONS DISCHARGED TO SKILLED NURSING FACILITIES BEFORE RETURNING HOME
Source: J Rehabil Med. 2024 Jun 20;56:35240. doi: 10.2340/jrm.v56.35240 (PMC11218674; doi:10.2340/jrm.v56.35240)
Supplement: EXPERIENCES OF CHAIN OF CARE AND REHABILITATION AFTER STROKE: A QUALITATIVE STUDY OF PERSONS DISCHARGED TO SKILLED NURSING FACILITIES BEFORE RETURNING HOME [file JRM-56-35240-s1.pdf]

Supplementary material has been published as submitted. It has not been copyedited, or typeset by Journal of Rehabilitation Medicine

Appendix S1

Interviewguide

How would you describe your rehabilitation from the hospital to today?

- What kind of training did you receive at the hospital?
- What kind of training did you receive at the skilled nursing facility?
- How do you feel that the various care facilities are linked together?
- Have you received any aids or adaptations at home?
    - If so, do you feel that they have been sufficient?
- What kind of training do you exercise today?
    - At the municipality?
    - In primary care?
    - Other training?
- Have you experienced the training as sufficient?
    - If not, what do you feel has been missing?
- How do you experience your involvement in setting goals and planning your rehabilitation after stroke?
- What do you find most troublesome today?
- How did you experience coming home?

Post-stroke Checklist

Since your stroke or last assessment:
1) Secondary Prevention
have you received any advice on health related lifestyle changes or medications for preventing another stroke?

2) Activities of daily life (ADL)
are you finding it more difficult to take care of yourself?
If yes, do you have difficulty dressing, washing and/or bathing? Do you have difficulty getting outside? Do you have difficulty preparing hot drinks and/or meals?

3) Oral health and Nutrition
Are you finding it more difficult to care for your oral health or nutrition? If yes, do you have difficulty taking care of your oral hygiene or have you lost your appetite? Have you difficulty to eat/swallow? Have you lost weight?

4) Mobility
are you finding it more difficult to walk or move safely from bed to chair? If yes, are you continuing to receive rehabilitation therapy?

5) Spasticity
do you have increasing stiffness in your arms, hands, and/or legs? If yes, is this interfering with activities of daily living?

6) Pain
do you have any new pain?

7) Incontinence
are you having more of a problem controlling your bladder or bowels?

8) Communication
are you finding it more difficult to communicate with others?

9) Mood
do you feel more anxious or depressed?

10) Cognition
are you finding it more difficult to think, concentrate, or remember things? If yes, does this interfere with activity or participation?

11) Fatigue
do you experience a mental fatigue that interferes with training and other activities?

12) Life after stroke
are you finding things important to you more difficult to carry out (e.g. leisure activities, hobbies, work, as well as relationships with loved ones)?

13) Relationship with family
has your relationship with your family become more difficult or stressed?

14) Other challenges?
Have you any other stroke related challenges that affect your recovery or causes you trouble?
